# Exosomes: A Key Piece in Asthmatic Inflammation

**DOI:** 10.3390/ijms22020963

**Published:** 2021-01-19

**Authors:** José A. Cañas, José M. Rodrigo-Muñoz, Marta Gil-Martínez, Beatriz Sastre, Victoria del Pozo

**Affiliations:** 1 Immunoallergy Laboratory, Immunology Department, Instituto de Investigación Sanitaria Fundación Jiménez Díaz (IIS-FJD), Avenida Reyes Católicos, 2, 28040 Madrid, Spain; jose.canas@fjd.es (J.A.C.); jose.rodrigom@quironsalud.es (J.M.R.-M.); marta.gilm@quironsalud.es (M.G.-M.); 2 CIBER de Enfermedades Respiratorias (CIBERES), Av. de Monforte de Lemos, 3-5, 28029 Madrid, Spain; 3 Faculty of Medicine, Universidad Autónoma de Madrid, 28029 Madrid, Spain

**Keywords:** asthma, extracellular vesicles, exosomes, biofluids, eosinophils, miRNAs

## Abstract

Asthma is a chronic disease of the airways that has an important inflammatory component. Multiple cells are implicated in asthma pathogenesis (lymphocytes, eosinophils, mast cells, basophils, neutrophils), releasing a wide variety of cytokines. These cells can exert their inflammatory functions throughout extracellular vesicles (EVs), which are small vesicles released by donor cells into the extracellular microenvironment that can be taken up by recipient cells. Depending on their size, EVs can be classified as microvesicles, exosomes, or apoptotic bodies. EVs are heterogeneous spherical structures secreted by almost all cell types. One of their main functions is to act as transporters of a wide range of molecules, such as proteins, lipids, and microRNAs (miRNAs), which are single-stranded RNAs of approximately 22 nucleotides in length. Therefore, exosomes could influence several physiological and pathological processes, including those involved in asthma. They can be detected in multiple cell types and biofluids, providing a wealth of information about the processes that take account in a pathological scenario. This review thus summarizes the most recent insights concerning the role of exosomes from different sources (several cell populations and biofluids) in one of the most prevalent respiratory diseases, asthma.

## 1. Introduction

Asthma is a heterogeneous airway disease with a complex inflammatory component. This disease is characterized by a dysregulated process that contributes to its maintenance, progression, and perpetuation. In this process, both resident cells (e.g., epithelial and endothelial cells, fibroblasts) and inflammatory cells (e.g., eosinophils, mast cells, T cells) interact with each other and secrete soluble mediators of inflammation, which drive disease pathogenesis [1,2,3].

**Figure 1:**
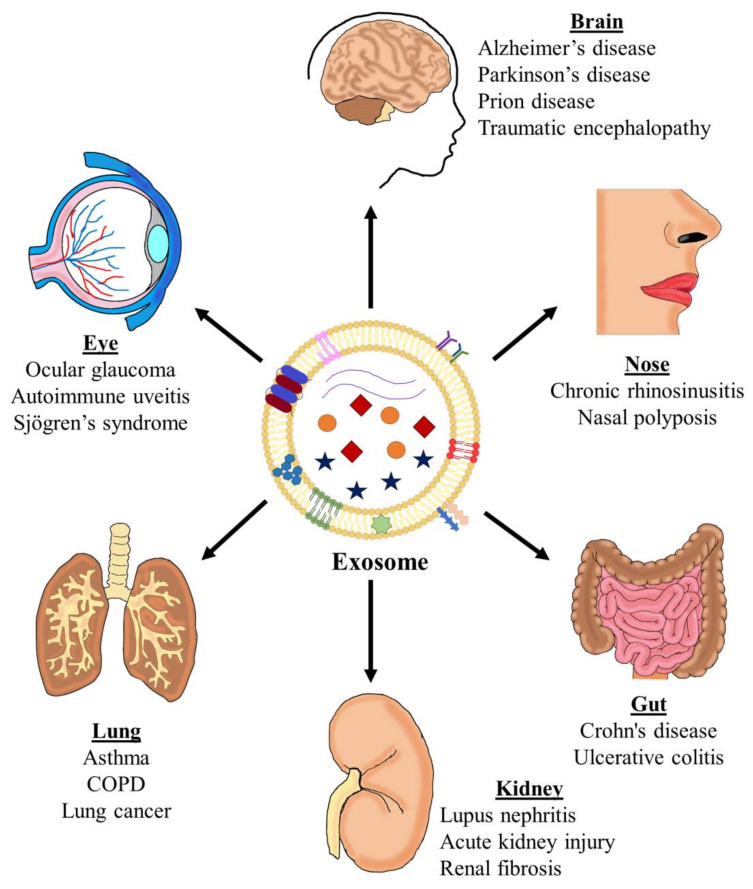
The implication of exosomes in inflammatory diseases. Exosomes have been described as modulators of different inflammatory diseases in different organs, including the brain (Alzheimer’s and Parkinson’s diseases), eye (ocular glaucoma), nose (chronic rhinosinusitis, nasal polyposis), lung (asthma, chronic obstructive pulmonary disease (COPD), and lung cancer), gut (inflammatory bowel disease), and kidney (lupus nephritis, acute kidney injury, and renal fibrosis). Figures inside and in the surface of the exosome represent some of their components. Inside: blue star: lipid mediators; exosome biogenesis proteins; violet lines: nucleic acids. In the surface: blue and red elipses: tetraspanins; green, red and pink structures: ceramide, phosphatidylserine, and sphingomyelin; violet and green: major histocompatibility complex (MHC)-I and -II, blue structure: integrin; pink structure; adhesion molecule; blue hexagons: cholesterol; green star: Rab proteins.

In recent decades, extracellular vesicles (EVs) have emerged as essential actors in intercellular communication through cell-to-cell contact or by shuttling different molecules, such as nucleic acids, lipids, and proteins. Exosomes can, therefore, significantly affect target cell function, resulting in the development of a pathological state [4]. For example, exosomes have been studied most extensively in association with different inflammatory pathologies, such as cancer and other infectious diseases [5,6], and also in asthma [7,8,9,10,11,12] (Figure 1).

In this review, we summarize current advances regarding the role of exosomes in the pathogenesis of asthmatic inflammation.

## 2. Extracellular Vesicles: Biogenesis and Composition

EVs are small vesicles released by donor cells into the bloodstream and body fluids, which can be taken up by recipient cells [13]. Though discovered decades ago, it has only recently become apparent that EVs play an important role in cell-to-cell (intercellular) communication and in the secretion of small soluble molecules into the extracellular environment (the secretome) as well as direct cell-cell contact [14].

EVs are heterogeneous spherical structures secreted into the extracellular microenvironment by almost all cells, both prokaryotic and eukaryotic [15]. Suspended in the aqueous nucleus or associated with the lipid casing is a wide repertoire of molecules contained and carried by EVs; these molecules include nucleic acids (DNA, mRNA, and small non-coding RNAs, such as microRNAs (miRNAs)), lipids, and proteins (cytokines, receptors, or their ligands) [16,17,18]. These vesicles are surrounded by a lipid membrane (phospholipid bilayer) enclosing the materials contained within, which are immersed in a small organelle-free cytosol [19,20]; this phospholipid bilayer creates a stable internal environment for biologically active components by protecting them from enzymatic degradation during transit throughout the extracellular environment [21].

The ability of EVs to carry a variety of nucleic acids, lipids, and proteins and, consequently, transfer this cargo to recipient cells influences various physiological and pathophysiological functions in these cells, causing EVs to have a significant impact on the phenotype of recipient cells [22]. For this phenotypic effect to occur, once EVs are released outside the donor cell, recipient cells can take these vesicles and trigger signaling events on the cell surface or be internalized by the cells, either through endocytosis or membrane fusion, which releases their contents inside the target cells, where they cause functional effects [23]. Among other diseases, EVs are implicated in inflammatory lung disorders, including asthma [7], chronic obstructive pulmonary disease (COPD) [24], and sarcoidosis [25], and maybe a universal spreader of inflammation.

**Figure 2:**
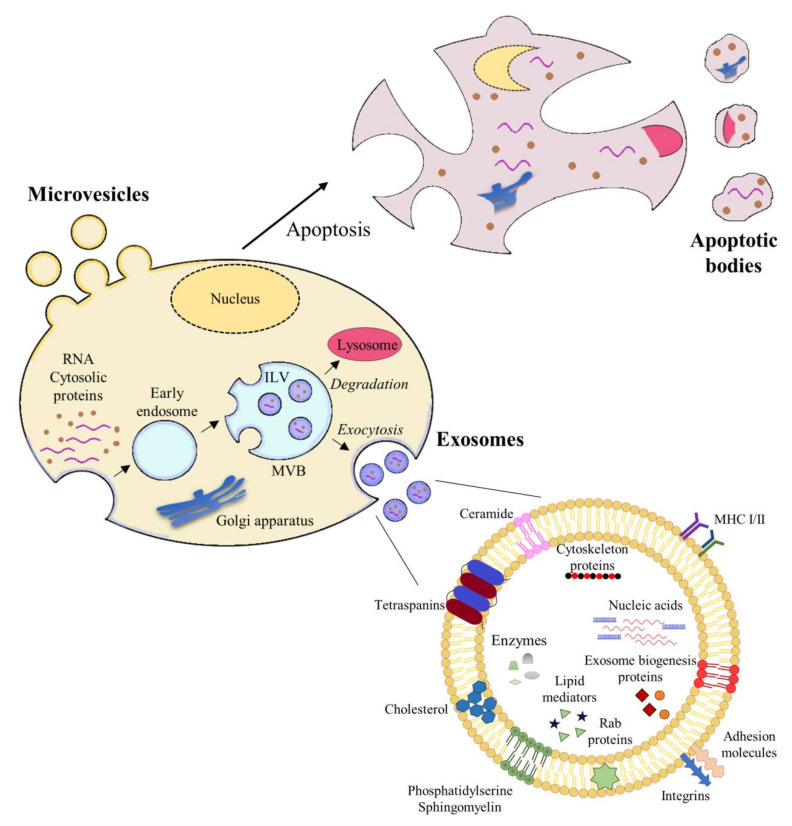
Extracellular vesicle biogenesis and exosome structure. Cells release several types of extracellular vesicles (EVs), including apoptotic bodies, microvesicles, and exosomes. Apoptotic bodies are large vesicles (1000–5000 nm in diameter), which are released from apoptotic cells. Microvesicles are medium-large EVs (100 nm to 1 µm in diameter), which originate directly from the cell membrane, followed by fission and release towards the extracellular space. Exosomes are small vesicles (30–150 nm) derived from endosomes. Maturation of early endosomes into late endosomes produces invaginations called intraluminal vesicles (ILVs). This endosome with ILVs is denominated multivesicular bodies (MVBs). These MVBs are able to fuse with lysosomes and degrade their cargo or with the plasma membrane to release ILVs towards the extracellular space. Exosomes are formed by a double lipid membrane and contain cytoskeletal proteins, tetraspanins, integrins, and adhesion molecules, and other proteins that reflect their endosomal biogenesis.

It should be noted that there have been discrepancies in the literature regarding the classification of EVs. Some studies divide them into two main subgroups: exosomes, which are vesicles released from multivesicular bodies (MVBs) by exocytosis, and ectosomes, vesicles assembled and released by the plasmatic membrane (PM) [26]. However, more recent studies categorize EVs according to their size, biogenesis, and release; these categories include large apoptotic bodies (> 1 µm); microvesicles (MVs), also called cellular ectosomes or microparticles, which comprise the intermediate fraction (200–1000 nm); exosomes, the smallest fraction (30–150 nm) [27] (Figure 2).

### 2.1. Apoptotic Bodies

Apoptosis, or “programmed” cell death, is an important mechanism of cell death in both normal and cancerous cells [28]. Whereas exosomes and MVs are secreted during normal cellular processes, apoptotic bodies are vesicles that are released only from cells undergoing this process of cell death (apoptotic cells) as products of apoptotic cell disassembly [29].

Apoptotic cells undergo a series of morphological changes, such as condensation of nuclear chromatin, fragmentation and degradation of internucleosomal DNA, nuclear and cellular organelle rupture (endoplasmic reticulum, Golgi and mitochondria, with the consequent release of cytochrome c), proteolytic cleavage of the cytoskeleton and focal adhesion complexes, phosphatidylserine (PS) externalization, alteration of key survival functions, blebbing of the PM, cell shrinkage, and commitment to the apoptotic phenotype. These changes ultimately package cell content in vesicles called apoptotic bodies (also called “apoptotic bullae” or “apoptotic vesicles”), ranging from 1–5 μm in diameter [30,31,32]. The term “apoptotic body” was coined by Kerr in 1972 [33].

Irrespective of the route to caspase activation, all pathways lead to the activation of the major effector caspases (3, 6, and 7), and these enzymes carry out much of the proteolysis seen during the apoptotic process [34].

During normal development, most apoptotic bodies are phagocytosed by macrophages and thus eliminated locally [35]. Clearance is mediated by specific interactions between certain molecules of the apoptotic cells’ membrane—due to specific changes in its composition—and recognition receptors on the phagocytic cells [36]. Translocation of PS to the external face of the PM, one of the hallmarks of apoptosis, and its subsequent binding to annexin V, recognized by phagocyte surface receptors, results in the digestion of “cell debris” [37]. In addition to this, oxidation of surface molecules, another well-characterized PM alteration, creates binding sites for thrombospondin (TSP) or complement protein C3b, also recognized by phagocyte receptors [36,38].

Therefore, the main protein markers of apoptotic bodies include, along with histones, annexin V, TSP, and C3b [39,40]. A notable distinction between apoptotic bodies and the other two major EV groups is that they also contain fragmented DNA and cellular organelles from their host cell [41].

### 2.2. Microvesicles (MVs)

Throughout the scientific literature, MVs have also been referred to as ectosomes and microparticles, among other names [42]. Ectosomes were first defined by Stein and Luzio, who observed ectocytosis and shedding of vesicles from the PM in stimulated neutrophils [43]. MVs are formed from the outward budding of the PM of the host cell surface [44]. The process that leads to MV generation starts with the formation of outward buds at specific sites of the membrane, followed by fission and subsequent release of the vesicle into the extracellular space [45].

MV biogenesis involves vertical trafficking of molecular cargo to the PM, molecular rearrangements, and the use of contractile actin-myosin machinery on the cell surface to allow for vesicle detachment [46]. The PM undergoes changes in lipid and protein composition and in Ca^2+^ levels, resulting in the recruitment and activation of calcium-dependent enzymes that are involved in disassembling the cytoskeleton and the exchange of lipids between the inner and outer leaflets of the membrane bilayer to maintain membrane asymmetry, thereby favoring budding and membrane abscission [47,48,49]. The asymmetric distribution of the PM is tightly regulated by aminophospholipid translocases [50]. In particular, externalization of the phospholipid PS occurs, which normally resides exclusively in the inner monoleaflet, which induces the formation of MVs [51]. Proteins that promote cytoskeleton contraction through actin-myosin interactions have been implicated in vesicle formation and cleavage [52]. Both cargo content and MV shedding are firmly regulated by several small GTPases, including members of the ADP-rybosilation factor (ARF; ARF1 and ARF6), Rab, and Rho (Rac1 and RhoA) families [53,54,55,56].

MVs are shed from the PM through direct outward budding, which defines their diameters and molecular compositions [57]. MVs are distinct from other EVs owing to the expressions of phospholipids and proteins on their surfaces [58]. Weerheim et al. determined that circulating MV membranes next to PS (3.63%) predominantly contained phosphatidylcholine (59.2%), sphingomyelin (20.6%), and also phosphatidylethanolamine (9.4%) [59]. Several studies have highlighted the fact that MVs contain a diverse population of proteins, including matrix metalloproteinases, glycoproteins, integrins, receptors, and cytoskeletal components; the main markers used to detect MVs are integrins, selectins, and cluster of differentiation (CD)40 [60,61,62,63]. The content of MVs also can include nucleic acids, particularly mRNA and miRNAs [64].

An important role in cargo selection seems to be that of the ARF6-regulated recycling pathway, which can regulate the inclusion of proteins, such as major histocompatibility complex (MHC) class I, β_1_ integrin receptors, vesicle-associated membrane protein 3 (VAMP3), and membrane type 1 matrix metalloproteinase (MT1MMP) [55].

### 2.3. Exosomes

The term exosome was coined by. Rose Johnstone to advance understanding of the biologic process underlying the transformation from a reticulocyte to a mature erythrocyte [65]. This nomenclature was adopted for vesicles released during reticulocyte differentiation as a consequence of MVB fusion with the PM [66].

Exosomes range in size from 30 nm to 150 nm and are formed within the cell by the inward invagination of late endosome membranes to form what has come to be known as MVBs [44]. The late endosome becomes a so-called MVB, comprising multiple vesicles, intraluminal vesicles (ILVs), and thus incorporating components of the cytosol [67]. These MVBs can either fuse with the lysosome if the content is destined for degradation or fuse with the PM, resulting in the release of the ILVs as exosomes into the extracellular space (Figure 2) [68].

ILV formation inside MVBs first requires reorganization of the endosome membrane, which is highly enriched in tetraspanins; second, the process must involve recruitment of the endosomal sorting complexes required for transport (ESCRTs) [69,70]. Four different ESCRTs have been designated, i.e., ESCRT 0, I, II, and III [71]. Although the ESCRT pathway is generally thought to be the main driver of exosomal biogenesis, the existence of ESCRT-independent exosome biogenesis has been shown [72], as seen in the involvement of sphingomyelinase activity, the implication of other lipids—cholesterol and phosphatidic acid—and the formation of these vesicles promoted by syntenin [73,74,75].

The Soluble NSF Attachment Protein Receptor (SNARE) protein complex has been implicated in the fusion of the MVBs with the PM, and the Ca^2+^-regulated vesicle-associated membrane protein 7 (VAMP7), a SNARE complex member, has been described as stimulating the release of acetylcholinesterase-containing exosomes [76]. Finally, in the release of ILVs as exosomes, a number of Rab GTPases, including RAB7, RAB11, RAB27A/B, and RAB35, are recognized as playing an important role [77].

Exosomes are typically composed of a lipid bilayer membrane and contain a luminal cargo that comprises proteins, DNA, RNA, peptides derived from lipids, surrounded by a lipid bilayer membrane (Figure 2). The phospholipid membrane contains lipids that bear the signature of the PM of the cell of origin, with high levels of cholesterol, sphingomyelin, ceramide, and detergent-resistant membrane domains, called lipid rafts [78,79]. The luminal content of exosomes predominantly includes cytosolic proteins derived from the donor cell [80]. Since exosomes originate from endosomes, proteins involved in MVB formation (e.g., Alix and tumor susceptibility geen [TSG]101), membrane transport, and fusion (e.g., annexins, flotillins, GTPases) are distinguishing proteins present on exosomes [81]. Another distinguishing feature of exosomes is the presence of tetraspanins, including CD9, CD63, CD81, and CD82 [82]. Other proteins present in exosomes include adhesion (e.g., integrins), antigen presentation (MHC class molecules), and heat shock proteins (HSP70, HSP90) [81].

Furthermore, many studies have shown the presence of nucleic acid cargo, which may include a variety of non-coding RNAs, including miRNAs and long non-coding RNA [82]. Other than different RNA species, exosomes also contain chromosomal and mitochondrial DNAs [83].

## 3. Exosomes in Biofluids in Asthmatic Inflammation

Asthma complexity and its multifactorial character have been mentioned previously. Given this complexity, when defining the disease, not only the cellular component is important, but also the soluble inflammatory microenvironment plays a key role in the development and evolution of asthmatic pathology.

Development of bodily fluid-extracted biomarkers would be a highly useful tool, as these would eliminate the need to employ more invasive procedures and tissue samples; however, a disadvantage is that fluids from the organism contain large amounts of aggregates and other components that pose contamination issue during isolation [84]. Pure EV isolation is mandatory to ensure that the results obtained are not confounded by contamination by viruses or other components [85]. Thus, there are multiple methods for the purification of EVs, based on the different characteristics of them. The methods present several advantages and disadvantages, and it is necessary to select the most appropriate method according to the specific characteristics of each sample (Table 1).

In the context of asthma, bronchoalveolar lavage and induced sputum will be the more representative biofluids of the lung environment, providing overall knowledge of the inflammatory composition of this specific microenvironment.

### 3.1. Bronchoalveolar Lavage Fluid

In bronchoalveolar lavage fluid (BALF), a widely used sample obtained from the lung, the main limitation to EV purification is the small volume of the specimen, creating a need to use an excellent purification method, for which there are several optimized techniques [100]. In this type of sample, the MVs (100–400 nm) are the main type of EVs, followed by exosomes [101].

Several studies have analyzed the effect of BALF-derived EV on the pathogenesis of asthma, using different approaches to evaluate this, from animal models to proteomics.

The first studies carried out by Prado et al. [102,103] focused on allergic murine models and demonstrated that BALF-derived exosomes inhibited specific immunoglobulin—(Ig)E and IgG1—and that pre-treatment with these exosomes also inhibited Th2 cytokines. In related findings, Shin et al. [104], using a murine model, observed that inhaled lipopolysaccharide (LPS)-induced BALF-derived EVs play an important role in the intercellular communication that takes place during the immune response and its possible dysfunction after inhaled LPS-containing allergens.

In human samples, the first studies with BALF exosomes [105] explored the phenotypic and functional characteristics of BALF-derived exosomes in asthma compared to others obtained from healthy subjects, simultaneously observing leukotriene (LT) biosynthetic capacity of these exosomes from asthmatic patients to leukotriene (LT) C4 (LTC_4_) and interleukin (IL)-8 release. BALF exosomes in the asthma context, therefore, might contribute to subclinical inflammation in airway epithelium. Recently, a study using mass spectrometry reported significant differences in the lipid composition of EVs between four groups studied (healthy, secondhand smoke (SHS)-exposed healthy, asthmatics, and SHS-exposed asthmatics), revealing that ceramides, ceramide-phosphates, phosphatidylglycerols, and sphingomyelins were altered based on pathology, and their abundance aided in discriminating between study groups [106]. Indeed, several studies about the influence of cigarette smoke on exosome production and composition have been developed [107]. These results point to a significant role for BALF-derived EVs, acting as elements to transfer active lipids. Besides lipid content, other manuscripts, such as the Rollet-Cohen et al. study [108], using a proteomic approach, demonstrated the different protein content of BALF exosomes from three different lung diseases (cystic fibrosis, primary ciliary dyskinesia, and asthma), observing different proinflammatory profiles.

EVs are an important transport element for multiple molecules, including miRNAs. Some asthmatic and allergic murine models have evaluated the role of these structures in the development and evolution of this disease, observing that some exert a proinflammatory effect, such as miR-21 [109], while another like miR-224, in an asthma murine model aggravated by particulate matter 2.5 (PM2.5), decreases the inflammation by targeting Toll-like receptor (TLR)2 and the reduction of Th17 inflammatory cells [110]. Moreover, Gon et al. [111] observed a higher amount of EVs in the airways in a house dust mite (HDM) murine model vs. control mice, identifying significant changes in the expression of 139 miRNAs from EVs and 175 miRNAs from lung tissues; a computational analysis revealed that 31 genes, including *IL13* and *IL5RA*, are putative targets of these miRNAs found to be up-regulated in EVs [111].

Several studies have been performed in humans, showing a relation of exosomes’ content of BALF with different clinical characteristics of the disease (Table 2), as the studies of Levänen [112] and Francisco-García [113].

Studies have even been conducted in the pediatric asthma population. Shi et al. [122] observed a higher expression of miR-26a, miR-146a, and miR-31 in BALF of asthmatic children compared to controls. In a manuscript from the same year, miRNA-let 7a, 7b, and 7c detected in BALF from asthmatic children were identified as biomarkers of asthma [123].

### 3.2. Induced Sputum

Nowadays, induced sputum is one of the biofluids with increasing applications, focusing on asthma research and diagnosis, as it is easily obtained and has a direct relationship with airway inflammatory status.

Globally, manuscripts about sputum in asthma have focused on miRNA contents and their functions in this disease, although in 2017, the first study was published in which exosomes of induced sputum were isolated from asthmatic patients [124].

In the same year, Maes et al. published their manuscript related to several miRNAs, severe and neutrophilic asthma [114] (Table 2); the group led by Liu found that miR-125b was downregulated in sputum from patients with eosinophilic asthma. They demonstrated an indirect role of this miRNA on the inhibition of goblet cell differentiation, being a potential candidate for improving therapeutic approaches for asthma [125]. In addition, miR-145 and miR-338 were also found together in several respiratory diseases like asthma, COPD, and asthma-COPD overlap syndrome (ACOS) [126]. Both miRNAs were more increased in supernatant than in peripheral blood; miR-145 was only elevated in asthma, while miR-338 was increased in all obstructive lung diseases analyzed. For both miRNAs, levels were higher in the supernatant of COPD and asthma patients than in controls. A study of cell-free sputum supernatants from allergic asthmatic patients showed a statistically significant reduction in the level of miR-155 compared to healthy subjects [127], leading the authors to suggest that the heterogeneous composition of sputum results in alternative miRNA expression levels. The authors further hypothesized that this downregulation of miR-155 might be linked to a lymphocyte dysfunction in the airways of these subjects. In a similar trend, miR-146a was lower in allergic asthmatics than in healthy controls. Recently, using sputum, an RNA sequencing and complex bioinformatics analysis showed a miRNA network associated with specific phenotypes of asthma [128]. Gomez et al. found a sputum miRNA network (particularly containing “nely” network module) associated with sputum neutrophilia and lymphocytosis, with a reduction of forced expiratory volume in 1 s (FEV_1_) percentage predicted and a decrease of the quality of life, just like increased hospitalizations in the previous year. This network was made up of 12 miRNAs, and among them, miR-223-3p was the miRNA most closely correlated with these clinical features, and these results were similar to those obtained previously by another group [114]. Moreover, classifying patients by their expression of “nely” miRNAs, the results revealed that subjects with asthma and these miRNAs had reduced FEV_1_ % predicted both before and after bronchodilation in a scenario with similar inhaled corticosteroids (ICS) doses; also, miR-223-3p expression levels were correlated with multiple features of severe asthma, bronchodilator response, and fractional exhaled nitric oxide (FeNO) levels [128].

### 3.3. Serum and Plasma

In the study of EVs in asthma pathology, other approaches may use serum or plasma. Just as in previous biofluids commented, EVs are the main transport elements of miRNAs and are resistant to RNase degradation.

Multiple miRNAs have been identified as playing a role in asthma pathogenesis and response to treatment, both in the adult and pediatric populations, which were measured in serum or plasma samples [129].

MiR-21 is one of the most widely studied miRNAs in several pathologies, including asthma, and it has been linked to several clinical parameters as eosinophil count, as in the manuscript of Elbehidy and colleagues [116] (Table 2). In a recent paper, another group confirmed elevated serum levels of miR-21 in patients with eosinophilic asthma compared to sera levels in healthy individuals [130]. A positive correlation was found between serum miR-21 and IL-4, confirming the role of this miRNA in Th2 activation and asthma pathogenesis. The authors also observed increased miR-155 expression in asthmatic sera; however, the absence of correlation with both IL-4 and miR-21 expressions indicated that the effect of both miRNAs on asthma pathogenesis is likely mediated by different pathways.

Prior to this manuscript, other groups demonstrated the role of miR-155 in both adult and childhood asthma pathology, like a manuscript of Karam and collaborators [117] in relation to miR-155 and let-7a (Table 2); similar results about let-7a have been previously detected [131]. Elevated levels of miR-155 in the serum of asthmatic children compared with those of the control group were observed by Liu and collaborators [132], who found a close association with the levels of indoor PM_2.5_ in the asthma group but not in the control group. Recently, this miRNA was considered an intracellular pro-inflammatory mediator of asthma, and the antagonism of miR-155-5p has been postulated to have corticosteroid-like effects on the treatment of asthma in a childhood asthmatic population [133]. As a result, miR-155 decreases glucocorticoid (GC)-induced NF-κB trans-repression. These authors predicted an improvement in lung function in the ICS treatment group with the combination of two miRNAs, that is, miR-155-5p and miR-532-5p, through a logistic regression model. In this line, Weidner and collaborators [134] found that miR-155 and miR-146 were differentially expressed in allergic asthmatic patients compared to a non-allergic asthma population, and this increase was observed when the subjects were using ICS. The two miRNAs share target genes involved in response to GCs and leukocyte regulation. Moreover, miR-223 and miR-374 showed a significant change in the non-allergic asthma group when blood eosinophil count was used as a classification parameter. These authors postulated that a combination of circulating miRNAs could be a tool that would aid in classifying asthmatic patients.

Similar to miR-155, other miRNAs, such as miR-16, may function as a biomarker to predict responses to therapy in asthma [118] (Table 2). In certain cases, miRNA expression levels differ based on asthma endotypes, such as neutrophilic or eosinophilic asthma [119,120] (Table 2), showing associations with different clinical parameters. In addition to miRNA functionality as predictors of treatment response, a recent paper from Fan and co-worker [135] demonstrated the role of miR-203a-3p in mechanisms linked to the development of classical asthma features. The authors of the study demonstrated that miR-203a-3p was able to modulate transforming growth factor-beta (TGF-β)1-induced epithelial-mesenchymal transition (EMT) through the Smad3 pathway. Yang et al. observed that miR-448-5p could affect TGF-β1-mediated EMT and pulmonary fibrosis in asthma [136]. Following this mechanistic approach, Du et al. [137] observed that miR-98-5p could be implicated in the development of bronchial asthma in a pediatric population through the decrease of the IL-13 expression.

However, due to the complex relationship between miRNAs and genes, not all miRNAs can be used as biomarkers. One solution to this problem may be to use combinations of several miRNAs or a specific miRNA profile, which may result in good sensitivity, specificity, and positive and negative predictive values. Our group, basing our work on an eosinophil miRNA profile, created a logistic regression model with three miRNAs (miR-185-5p, miR-144-5p, and miR-1246) to better discriminate between asthma and healthy subjects; indeed, a Random Forest model created with miR-185-5p, miR-320a, and miR-144-5p was capable of separating healthy individuals from asthma patients and, within the disease group, classified each one in terms of disease severity [138]. Findings from our study suggest that the miRNA profile detected in eosinophils could be used as a diagnostic tool for asthma in serum and rank patients according to severity. In this line, some manuscripts about the prediction of exacerbations have been published [121] (Table 2). However, miRNAs contained in EVs are not the only good element to characterize asthma or predict disease course or treatment response. This year, several manuscripts have probed the importance of serum EVs analysis to understand other parameters that can exert an effect on asthma pathology. Lee et al. [139] used EVs from the serum of healthy and asthmatic individuals to extract DNA and study, by means of metagenomic analysis, the microbial composition and its relation to clinical characteristics of asthma. The authors obtained a bacterial composition that was significantly different between the two groups, creating a diagnostic model based on these differences with good predictive values (sensitivity: 0.92, specificity: 0.93). They, therefore, demonstrated the important role of the microbiome as a potential diagnostic marker of asthma, employing the serum. Kim et al. [140] focused their attention on bacterial EVs IgG antibody titers in serum, observing that in asthma and COPD patients, these titers were higher than in healthy controls, postulating that these values could be used as a diagnostic tool for lung disease.

As we have previously reported, there are studies analyzing the relationship between exosome production and various clinical parameters; however, the effect of age on exosome synthesis is a field yet to be developed, with some existing data concluding that EVs in plasma decrease with age [141].

All these results and data demonstrate that more research is needed to elucidate the roles that EVs and their contents, such as miRNAs, can play as biomarkers or predictors in the therapeutic response in asthma. Likewise, studies comparing EVs with other biomarkers that are already well characterized and available are needed.

## 4. Exosomes from Eosinophils as Key Cells in Asthma

Eosinophils are innate immune cells that have been widely associated with asthma pathophysiology [142]. This end-stage granulocyte derived from bone-marrow progenitor cells can affect lung physiology, the inflammatory focus in asthma, driven by diverse mediators [143]. The development, survival, and migration of eosinophils are due to actions of key cytokines and chemokines of type 2 (T2) immune response as IL-5, IL-4, RANTES (C-C chemokine ligand [CCL]5), and eotaxins (eotaxin-1 (CCL11), eotaxin-2 (CCL24), and eotaxin-3 (CCL26)), which are recognized by receptors (IL-5Rα and C-C chemokine receptor [CCR]3) in the eosinophils, activating their functions [144,145,146].

The array of T2 cytokines, which induces eosinophils’ recruitment and activation in asthma pathophysiology, is released by diverse cell types, including Th2 lymphocytes [147] and type 2 innate lymphoid cells (ILC2s) [148]. ILC2s are the first source of T2 cytokines when recruited by alarmins released by the lung epithelium (IL-25, IL-33, and thymic stromal lymphopoietin (TSLP)) after an allergen encounter [149,150].

Eosinophils recruited to the lungs are triggers of asthma hallmarks, which include airway remodeling (increase in smooth muscle mass and epithelial desquamation), mucus hypersecretion, and local inflammation. This array of immuno-structural regulations is done by the eosinophil granule proteins, which contain variable enzymes, compounds, and cytokines. Their granules and vesicles can be studied by electron microscopy [151,152] and contain receptors for eotaxin, interferon-gamma (IFNγ), and CCR3, making them autonomously competent organelles capable of releasing their contents upon ligand binding [153] by exocytosis, vesicle-mediated piecemeal degranulation [154,155,156], or by eosinophil cytolysis [157]. The composition of protein granules consists of enzymes that cause epithelial damage, such as eosinophil cationic protein (ECP), eosinophil peroxidase (EPO), eosinophil-derived neurotoxin (EDN), or major basic protein (MBP) [158,159,160].

Eosinophils are able to release other molecules involved in remodeling, including metalloproteinases like matrix metalloproteinase 9 (MMP-9) [161], molecules affecting inflammation, such as nitric oxide (NO), lipid mediators like LTs (promote autocrine eosinophil migration and survival) [162], and reactive oxygen species (ROS) that contribute to airway injury [163]. Furthermore, eosinophils have the capacity of rapidly releasing very diverse preformed cytokines both from T1 and T2 immune pathways (IL-4, IL-6, IL-10, IL-12, IL-13, IFNγ, and tumor necrosis factor-alpha (TNFα)), due to their storage in granules and vesicles, which facilitates fast release in response to different stimuli, such as vesicle transported-IL-4 or eotaxin [158,164,165,166,167,168].

Equally important are the eosinophil extracellular DNA traps (EET) (from the nuclear or mitochondrial origin), consisting of the release of DNA from the cell as a physical net with which to capture pathogens [169]. Sometimes, this liberation of extracellular DNA traps causes eosinophil death (EETosis) [170,171]. In fact, eosinophils from severe eosinophilic asthma have displayed an increased percentage of eosinophil extracellular traps when treated with LPS and IL-5. These traps can autocrinally promote eosinophil degranulation, inducing further inflammation in the airways of severe asthmatics [172].

In the last few years, a new eosinophil derivate has been studied due to its role in asthmatic disease: the exosomes. The road to the discovery of eosinophils’ capability to release exosomes began in 2002 when the tetraspanin CD63 was described inside eosinophils as being involved in piecemeal degranulation after stimulation with IFNγ [173]. A few years later, in 2009, Akuthota et al. showed that CD9, another exosomal marker, was expressed on the surface of eosinophils and that it colocalized with MHC class II in the detergent-resistant membrane microdomains (DRMs) after stimulation with granulocyte-macrophage colony-stimulating factor (GM-CSF). These two proteins give eosinophils the capacity to function as antigen-presenting cells [174].

Given that the multivesicular body marker CD63 is inside eosinophils, that eosinophils express CD9 on their surfaces, and that other cells of the immune system are capable of releasing exosomes [7], it is not unreasonable to consider that eosinophils are able to secrete them, something which was speculated back in 2012 [175].

The theory was confirmed in 2015 when Mazzeo et al. [11] described the presence of MVBs inside eosinophils. Using antibodies against the endosomal marker CD63 and the multivesicular body marker lysobisphosphatidic acid (LBPA), the authors were able to detect colocalization of both proteins in eosinophilic granules by confocal microscopy and transmission electron microscopy (TEM). Interestingly, the addition of IFNγ, a well-known stimulator of granule mobilization, caused enhancement of CD63 and LBPA expressions in the PM. This confirmed vesicle fusion from the cytoplasm to the membrane to release their contents. This mobilization was observed by time-lapse fluorescence microscopy and flow cytometry, showing how the cytoplasmic levels of CD63 and LBPA decreased upon IFNγ stimulation.

To characterize exosomes from eosinophils, the authors purified exosomes from eosinophils of asthmatic or healthy subjects. Validation of exosomal markers was performed by Western blot (Alix, CD63, and CD9) and TEM (Alix, CD63), and exosomes were measured by nanoparticle tracking analysis (NTA). Results showed that exosomes indeed expressed specific markers and had the expected exosomal size. Furthermore, NTA showed that non-stimulated eosinophils from asthmatics were able to produce higher amounts of exosomes when compared to eosinophils from healthy sources, while IFNγ stimulation yielded no differences for the secretion of exosomes of asthmatics compared to healthy individuals. Finally, using Western blotting, the authors confirmed the presence of the eosinophil enzymes EPO, MBP, and ECP in exosomes derived from eosinophils, and the quantity of these proteins was similar between exosomes from asthmatic and healthy subjects. Together, these results confirmed the capacity of eosinophils to release exosomes, further showing that exosome secretion is higher in eosinophils from asthmatics, which may be related to the direct correlation found in another study between the EV concentration in the asthmatic airway and peripheral eosinophilia, meaning that the exosomes may play a role in asthma pathology [11].

A year later, an independent research group corroborated Mazzeo et al.’s results. Using techniques, such as TEM, nanoscale flow cytometry, and protein electrophoresis, the authors were able to detect the exosomes released by eosinophils, confirming their size and presence of CD63 and CD9 [176]. Using TEM, they observed an increase in MVB release after treatment with CCL11, and even higher with TNFα, compared to untreated individuals. Additionally, stimulation increased the number of MVB-producing eosinophils from 50% to 90–100%, while MVBs released by the effect of TNFα were smaller in size than those released following administration of CCL11 or when untreated. The results of the study also highlighted the importance of marker selection for MVBs, around 50% of MVBs were stained for CD63 and only 15% for CD9, so seemingly CD63 might be a better marker for MVBs. Finally, the authors confirmed MVB formation after stimulation by annexin-V and reported that MBVs were not apoptotic bodies, as evidenced by TUNEL assay in confocal microscopy [176].

Experimental procedures performed by Cañas et al. showed that exosomes released from eosinophils were taken up by eosinophils themselves. Exosomes from asthmatics were able to autocrinally increase the production of NO (colorimetric assay), while exosomes isolated from both healthy and asthmatic subjects induced ROS synthesis (flow cytometry intracellular staining) [177]. Proteomic mass spectrometry analysis was carried out in order to determine exosome contents, revealing that the contents were similar to those usually secreted by eosinophils, including ECP, MBP, EPO, asthma-related proteins like periostin [178], and others related to migration, adhesion, cell signaling, redox, inflammation, or metabolism; no differences were found to reflect the asthmatic or healthy origin of these contents. Basal eosinophil apoptosis was initially higher in eosinophils from healthy subjects, and the addition of exosomes isolated from healthy or asthmatic subjects did not cause any effect. Nonetheless, adhesion, chemotaxis, and chemokinesis were increased by exosomes obtained from asthmatics. This enhancement of adhesion was accompanied by upregulation of intercellular adhesion molecule (ICAM)-1 and integrin α_2_ on the surfaces of eosinophils treated with asthmatic exosomes, augmenting eosinophil inflammation [179,180].

All these results confirmed that exosomes derived from asthmatics’ eosinophils could upregulate eosinophils’ own functions, probably due to their protein content quantity and due to the enhanced capacity of asthmatic eosinophils to release exosomes [11,177].

When exosomes derived from asthmatic or healthy eosinophils were taken up by airway structural cells (small airway epithelial cells (SAECs) and bronchial smooth muscle cells (BSMCs)), several changes in their behavior were induced [12]. Specifically, wound healing capacity was delayed, while an increase in apoptosis (measured by annexin-V and TUNEL assay) was observed in SAEC cultured after 24 h with exosomes from asthmatics. Additionally, gene expression of the *TNF*, *CCL26*, and *POSTN* was upregulated by the addition of asthmatic exosomes at different time points, depending on whether there was an epithelial wound or not. Many of these phenotype changes result from the effect of asthmatic exosomes on epithelial phosphorylated protein kinase B (pAKT) and phosphorilated signal transducer and activator of transcription (pSTAT3)measured by Western Blot, with a reduction of both proteins at 24 h and enhancement at 48 h.

In contrast, BSMCs treated with asthmatic exosomes showed increased proliferation at 72 h due to an enhancement of phosphorylated extracellular regulated kinase (pERK) protein levels and underwent an increase in the expression of the proangiogenic and chemotactic genes *VEGFA* and *CCR3*.

**Figure 3:**
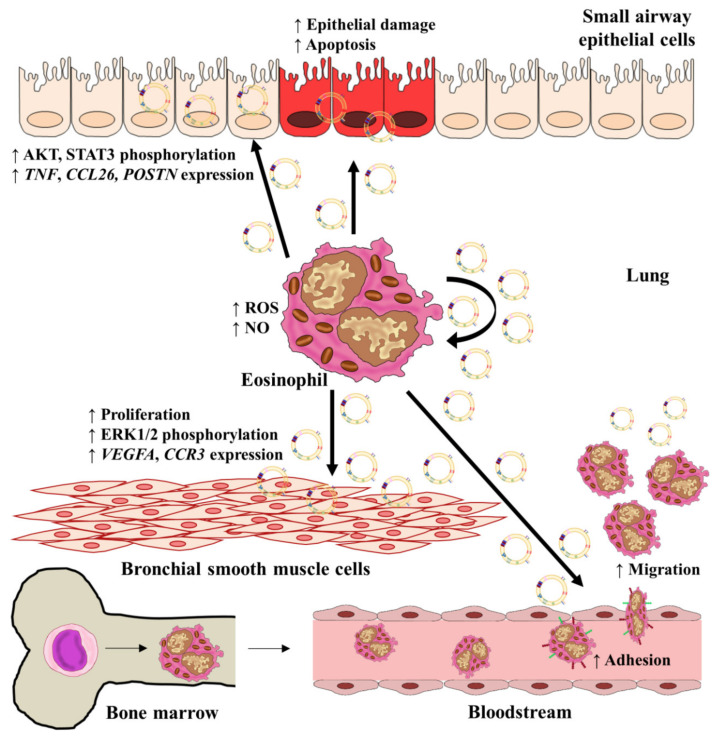
Specific roles of eosinophil-derived exosomes in asthmatic inflammation. Exosomes released by eosinophils are able to alter several functions associated with asthmatic pathology on both eosinophils themselves and structural lung cells, such as small airway epithelial cells and smooth bronchial muscle cells. Exosomes can increase eosinophil adhesion and migration and the release of ROS and NO, contributing to tissue damage. Moreover, exosomes augment BSMC proliferation and SAEC injury, increase the expression of several genes implicated in asthmatic inflammation and remodeling (*TNF*, *POSTN*, *CCL26*, *POSTN*, *VEGFA*, and *CCR3*), and alter some pathways implicated in asthma, including MAPK and JAK/STAT. Abbreviations. AKT: protein kinase B; STAT3: signal transducer and activator of transcription 3; TNF: tumor necrosis factor; CCL26: C-C chemokine ligand 26; POSTN: periostin; ROS: reactive oxygen species; NO: nitric oxide; ERK: extracellular regulated kinase; VEGFA: vascular endothelial growth factor A; CCR3: C-C chemokine receptor 3.

All these results demonstrate the role of exosomes from asthmatic eosinophils as key molecules, contributing to structural lung-cell activation and airway remodeling [12]. Therefore, we can conclude that exosomes from eosinophils are indeed autonomous molecules that are able to modulate and enhance the pathophysiology of asthma acting on eosinophils and also on structural lung cells alongside the rest of the compounds released by these immune cells (Figure 3).

## 5. Exosomes from Other Cellular Populations Implicated in Asthma Pathology

As remarked previously, exosomes can be found in multiple localizations and can be released by a variety of cell types. This fact draws a resemblance between exosomes and their origin cells in terms of proteins and nucleic acids. Airway inflammation in asthma pathogenesis is driven by several effector cells, including T and B lymphocytes, macrophages, mast cells, eosinophils, and structural lung cells (epithelial and smooth muscle cells) [181]. These cells exert their functions either directly or indirectly, via exosomes and EVs [4]. This part of the review, therefore, focuses on the most recent findings related to exosomes released by the principal effector cells that participate in inflammation of the asthmatic disease.

Peripheral blood mononuclear cells (PBMCs) have a round nucleus and comprise lymphocytes (T and B) and monocytes/macrophages, principally.

It is known that T lymphocytes play a major role in the inflammatory response of asthma, leading to tissue remodeling and airway hyperresponsiveness; moreover, it is accepted that B cells exert important functions in the adaptive immune response to this disease [182]. Exosome release from T cells has been described [183]. Several studies by Shefler et al. showed the role of EVs from these cells in the inflammatory lung response [184,185], describing their role in mast-cell activation and degranulation, releasing cytokines (IL-8 and oncostatin M) [185], linking the miR-4443 to some of these processes [184]. They demonstrate the activation of inflammatory cells through exosomes in different locations.

B cells were first reported to release exosomes more than twenty years ago [86]. B cell-derived exosomes carry specific molecules of antigen-presenting cells, including MHC class I and II, integrins, and costimulatory molecules (CD40, CD81, and CD86) [86,186]; these characteristics allow the exosomes to be functional units for antigen presentation, inducing T cell responses, modulating their proliferation and the release of IL-5 and IL-13 cytokines. Although exosomes can conduct themselves as immunostimulatory molecules, the immunoregulatory role of these nanovesicles has also been described [187].

On the other hand, macrophages and monocytes also play several roles in asthmatic inflammation [188]. In asthma, the release of Th2 cytokines, including IL-4 and IL-13, allows macrophage polarization towards an M2 phenotype [189]. Regarding exosomes, several years ago, it was also described that macrophages could release these nanovesicles [190]. Some data could indicate that exosomes from macrophages might be implicated more in T1 immune response than in T2 response [190,191,192]. However, Esser et al. demonstrated that these macrophage-derived exosomes carried different enzymes implicated in LT biosynthesis, particularly LTB_4_, which is a potent chemoattractant agent for eosinophils and neutrophils [192]. Moreover, a recent study demonstrated that exosomes from M2 macrophages induced the differentiation of ILCs progenitors to ILC2, a potent source of immune effector cytokines in asthma [193].

Although these data demonstrate the role of exosomes from PBMCs in inflammation, more studies are necessary to elucidate the specific mechanisms of these nanovesicles on inflammatory airway diseases.

Other cell populations that could produce exosomes are polymorphonuclear cells (PMNs), which primarily comprise neutrophils, eosinophils, basophils, and mast cells. The functions of these cells in allergic and severe asthma inflammation have been widely described [145,194,195].

Neutrophils have been associated with non-T2 response and severe asthmatic phenotype [196] and are able to produce exosomes with a potential role in asthma. In 2016, Vargas et al., using an equine model of asthma, demonstrated that neutrophil exosomes contributed to airway remodeling and tissue inflammation by modulating apoptosis and proliferation of smooth muscle cells [197]. In 2016, Butin-Israeli et al. observed that EVs from neutrophils possessed MMP-9 activity, degrading proteins of the tight junctions and, consequently, breaking down epithelial unions [198]. Recently, an exhaustive study on the roles of neutrophil-derived exosomes in airway inflammation conducted by Genschmer et al. [199] described that exosomes from neutrophils that contained elastase had proteolytic activity in the extracellular matrix, contributing to inflammation, epithelial damage, and airway remodeling. Moreover, they observed that the elastase contained inside of exosomes had more powerful effects than elastase-free.

Mast cells are another granulated cell type involved in asthma pathology [200], and some authors have studied the effect of mast-cell exosomes in the modulation of allergic inflammatory responses [201]. Various groups have demonstrated different ways in which mast-cell exosomes can act in the context of asthmatic disease, finding that exosomes could act by interacting with cells, such as airway smooth muscle [202], exert their inflammatory functions without contact [203], or act as immunoregulatory units [204].

Basophils are a population of granulated leukocytes that comprise 0.5–1% of peripheral blood white cells and are involved in immune responses [205]. There is a lack of evidence that basophils can release exosomes [205], although some authors have described that they could release granules that resemble exosomes [206].

### Exosomes Released by Other Cells Implicated in Asthma Inflammation

Aside from immune mediator cells, other cell types are implicated in asthma pathogenesis and airway remodeling, such as structural lung cells, platelets, and mesenchymal stem cells (MSCs).

Structural lung cells, including airway epithelial cells (AECs) and airway smooth muscle cells (ASMCs), play pivotal roles in asthmatic disease and inflammation [207,208]. T2 cytokine-stimulated exosomes presented an increase in nitric oxide synthase 2 (NOS2), and exosomes from T17-stimulated epithelial cells showed a capacity for neutrophil chemotaxis. Kulshreshtha et al. demonstrated in a murine model that stimulated IL-13 epithelial cell-derived exosomes induced infiltration and proliferation of macrophages in the lungs [207]. However, most articles about exosomes from ASMCs in asthma are based on the effect of exosomes from other cell types in ASMCs [12,197,202].

On the other hand, although the roles of microparticles from platelets in airway hyperresponsiveness and bronchial remodeling have been described [209,210], unfortunately, no studies have been conducted on platelet-derived exosomes.

Finally, many studies about MSCs-derived exosomes in asthma have been performed. MSCs are pluripotent stromal cells that can reduce airway inflammation in asthma, thus increasing the proliferation of T regulatory cells (Treg) [211]. Recent studies showed the immunomodulatory role of MSC-derived exosomes in asthma pathogenesis [212] as well as their role in airway inflammation [213], demonstrating that exosomes from MSCs produce attenuation of airway inflammation, showing a similar effect to MSCs. This effect is produced by an increase of Treg cells and immunosuppressive cytokines, including IL-10 and TFG-β [213]. The role of the immunosuppressive effect of MSC-derived exosomes has also been studied in other inflammatory and non-inflammatory pathologies. It has been observed in eye diseases, where MSCs-derived exosomes promote regulation of Treg, providing an immunosuppressive microenvironment in the inflamed eyes [214]. Besides, immunosuppression promoting by MSCs exosomes has been observed in allergic contact dermatitis [215], diabetes [216], and in diseases with liver injury [217] by influencing Treg proliferation. Recently, Riazifar et al. demonstrated that exosomes of MSCs reduced pro-inflammatory cytokine levels and promoted Treg expansion in an experimental autoimmune encephalomyelitis model of multiple sclerosis [218]. On the other hand, different studies showed the capacity of exosomes to modulate the functions of other immune cells, such as dendritic cells [219]. Another study by Fang et al. in 2020 showed that small EVs from MSCs modulated ILC2 functions in vitro, decreasing allergic airway inflammation in mice through the delivery of miR-146a-5p [220].

The use of MSCs-derived exosomes as therapeutic tools has been widely studied and has shown promise in the field of regenerative medicine. Numerous studies have explored the therapeutic effects of exosomes from MSCs on neurological, immunological, and cardiovascular diseases [221]. Specifically, in asthma, several works have addressed this topic [222]. In 2010, Porro et al. demonstrated that exosomes from MSCs had the ability to accelerate lung tissue repair and wound healing, which could be used to alleviate asthmatic airway remodeling [223]. Other studies demonstrated that MSC-derived exosomes could mitigate airway inflammation in asthma pathology by increasing the proliferation of Treg, promoting an increase of anti-inflammatory cytokine production and immunosuppressive capacity [213]. Furthermore, exosomes from human adipose tissue-derived MSCs are able to inhibit airway hyperresponsiveness and airway inflammation in a mouse model of ovalbumin-induced asthma [224].

All of these studies underscore the importance of exosomes from any cell type in asthma and inflammation, as well as in the immunoregulation of this disease. As a result, exosome research is a promising field that may aid in understanding asthmatic diseases and in the search for novel therapies.

## 6. Conclusions

In the last decades, EVs have emerged as important and revolutionary elements of intercellular communication, both in physiological processes and in various pathologies. Their capacity to transport multiple elements, such as proteins, lipids, and nucleic acids, among which miRNAs are found, makes them highly relevant factors in multiple processes. All these characteristics turn these intracellular generating elements into factors of great relevance to understand, in many cases, the development of diverse diseases as well as many of their physiopathogenic mechanisms, being able to be keys to find new therapeutic approaches. The study of EVs in specific biological samples, like induced sputum or serum likewise diverse biofluids, can aid in getting knowledge about diverse pathologies, like asthma.

## Figures and Tables

**Table 1:** Methods to isolate extracellular vesicles (EVs).

Method	Methodology	Advantages/Disadvantages	Ref.
Differential centrifugation	* Stepwise manner. * Sequential centrifugations, increasing the centrifugation speed.	- Low cost, large quantities of the solution, absence of chemicals. - Complexity, equipment (ultracentrifuge), and efficiency depend on the type of rotor.	[39,86,87]
Density gradient centrifugation	* Initial samples are EVs, partially isolated by differential centrifugation. * Use of solutions of sucrose, iohexol, or iodixanol.	- Pure preparations, no contamination with viral particles, absence of chemicals. - Complexity, equipment (ultracentrifuge), loss of sample.	[39,88,89]
Chromatography	* Filtration through columns of porous smaller than EV of interest	- Rapid isolation, preservation of vesicle integrity. - Limitations of sample volume, specialized equipment, complexity.	[90,91]
Ultrafiltration	* Use of porous membranes to trap molecules with a specific size through successive steps to obtain EVs with the desired size. * Based on size and mass.	- Simplicity, processing of many samples, lack of limitations on sample volume. - Sample contamination by proteins, loss of sample by filter plugging	[92,93]
Precipitation by chemicals	* Use of organic solvents, polyethylene glycol, sodium acetate, or protamine.	- Relatively quick, able to be used in a wide range of samples. - Contamination with non-EV proteins, retention of chemicals, long processing time.	[94,95,96,97]
Precipitation by polymers	* Commercial kits * Use of super hydrophilic polymers solutions, or PEGs. * Diminished the solubility of EVs and generation of a pellet precipitate.	- Simple procedure, no need for additional equipment. - Usually costly, not be good for large samples of EVs, high concentration of impurities.	[39,90]
Precipitation by protein surfaces (immunoassay)	* Immunoprecipitation. * Magnetic beads coated with antibodies for common EV surface proteins, such as CD63, CD9, and CD8. * Use after a centrifugation method for isolation.	- High purity and selectivity. - High cost, selectivity may be too high, difficulties for detachment antibodies and to analyze the intact vesicles.	[98,99]

**Table 2:** Summary of data concerning miRNAs detected in different biofluids linked to clinical parameters.

Sample	Groups of Study	Results	Function/Effects	Ref.
Bronchoalveolar lavage fluid	* Healthy and asthma individuals. * Severe asthma patients.	- 24 miRNAs differentially expressed from 894 miRNAs evaluated. - Prominent role of the let-7 family, especially miR-200. - Deficient loading of miRNAs into their nanovesicles. These miRNAs generated a network.	- Downregulated in asthma group. Correlated with airway remodeling. - MiRNAs network associated with worsened lung function and increased eosinophilic and neutrophilic inflammation.	[112] [113]
Induced sputum	* Healthy, mild-to-moderate and severe asthma patients. * Healthy and asthmatic patients.	- Higher expression of miR-629-3p, miR-223-3p, miR-142-3p. - Used epithelium, sputum, and plasma samples. - In sputum, miR-221-3p correlates with eosinophils. - Increase of miR-221-3p after 4 weeks of inhaled corticosteroids compared to baseline.	- Related to neutrophilic inflammation. - Biomarker for airway eosinophilic inflammation; moreover, being an element of airway inflammation improve after treatment.	[114] [115]
Serum and/or plasma	* Asthmatic and healthy children. The author sub-classified asthmatics children into two groups, steroid-resistant and steroid-sensitive.	- Serum miR-21 level was increased in asthmatics vs. healthy as well as in steroid-resistant patients compared to steroid-sensitive patients. - Positive correlation with blood and sputum eosinophil count and inversely correlated with FEV_1_.	- MiR-21 could be a severity biomarker in asthma pathology.	[116]
	* Asthmatic and healthy children.	- Higher levels of miR-155 in plasma from asthmatic patients and decreased levels of let-7a. - MiR-155 presented a direct correlation with IL-13 levels and an inverse correlation with FEV_1_ and FVC. Let-7a correlated positively with FEV_1_ and FVC and inversely with IL-13 expression.	- MiR-155 and let-7a showed opposite results. MiR-155 could be a biomarker of worsened lung function.	[117]
	* Salmeterol-sensitive and resistant asthmatic patients. * Neutrophilic asthma patients and healthy subjects. * Asthma and healthy individuals. * Pediatric asthma cohort.	- Serum MiR-16 levels present a significant negative correlation with FEV_1_. - MiR-199a-5p was increased in plasma and sputum of patients with neutrophilic asthma. Negative correlation with pulmonary function. - Strong inverse correlation between plasma miR-181b-5p and airway eosinophilia. - Increase of miR-181b-5p levels after ICS treatment. - Evaluation of 754 miRNAs in serum from 153 asthmatic children. - 12 miRNAs had significant odds ratios for exacerbation, the most significant being miR-206. - miR-146b, miR-206, and miR720 combination, alongside the exacerbation clinical score, presented a predictive power with an area under the ROC curve (AUC) of 0.81.	- Mir-16 may predict response to salmeterol with an AUC value of 0.99, being a potential biomarker in response to treatment. - Plasma miR-199a-5p could be a marker of neutrophilic asthma and poor lung function. - Biomarker for airway eosinophilia. - Using the logistic regression model created with three miRNAs, it may be possible to predict exacerbations.	[118] [119] [120] [121]

## Data Availability

Not applicable.

## Abbreviations

The following abbreviations are used in this manuscript:

BALF	bronchoalveolar lavage fluid
BSMCs	bronchial smooth muscle cells
EMT	epithelial-mesenchymal transition
EVs	extracelular vesicles
ICS	inhaled corticosteroid
ILC	innate lymphoid cell
ILVs	intraluminal vesicles
MSCs	mesenchymal stem cells
MVBs	multivesicular bodies
PM	plasmatic membrane
PMNs	polymorphonuclear cells
Treg	regulatory T cells
SAECs	small airway epithelial cells
